# Probiotics and Fecal Transplant: An Intervention in Delaying Chronic Kidney Disease Progression?

**DOI:** 10.3390/clinpract13040080

**Published:** 2023-08-01

**Authors:** Harshavardhan Sanekommu, Sobaan Taj, Rida Mah Noor, Muhammad Umair Akmal, Reza Akhtar, Mohammad Hossain, Arif Asif

**Affiliations:** 1 Department of Medicine, Jersey Shore University Medical Center, 1945 NJ-33, Neptune City, NJ 07753, USA; sobaan.taj@hmhn.org (S.T.);; 2 School of Medicine, Eastern Campus, International University of Kyrgyzstan-International, Bishkek 720007, Kyrgyzstan; ridamahnoor926@gmail.com; 3 Kazakh National Medical University, 050012 Almaty, Kazakhstan; umairakmal02@gmail.com; 4 Department of Gastroenterology, Jersey Shore University Medical Center, 1945 NJ-33, Neptune City, NJ 07753, USA

**Keywords:** prebiotics, probiotics, fecal microbiota transplantations, chronic kidney diseases

## Abstract

Chronic kidney disease (CKD) is a global health challenge affecting nearly 700 million people worldwide. In the United States alone, the Medicare costs for CKD management has reached nearly USD 80 billion per year. While reversing CKD may be possible in the future, current strategies aim to slow its progression. For the most part, current management strategies have focused on employing Renin Angiotensin Aldosterone (RAS) inhibitors and optimizing blood pressure and diabetes mellitus control. Emerging data are showing that a disruption of the gut–kidney axis has a significant impact on delaying CKD progression. Recent investigations have documented promising results in using microbiota-based interventions to better manage CKD. This review will summarize the current evidence and explore future possibilities on the use of probiotics, prebiotics, synbiotics, and fecal microbial transplant to reduce CKD progression.

## 1. Introduction

Dysbiosis, defined as an imbalance in the microbiota, has been linked to various immune-mediated conditions [1]. In chronic kidney disease (CKD), dysbiosis is a consequence of the retention of metabolic waste products, uremic toxins derived from gut bacteria, which is exacerbated by concomitant medication use, poor nutrition, and comorbidities. In turn, dysbiosis contributes to the progression of CKD by promoting harmful immune responses, microbial metabolites, the activation of the Renin–Angiotensin System (RAS), and the disruption of the gut barrier (Table 1) [2]. When the gut barrier is penetrated, aerobic bacteria grow and produce uremic toxins such as para-cresol sulfate (PCS), indoxyl sulfate (IS), and trimethylamine N-oxide (TMAO). These toxins are harmful to the renal tissue and contribute to mortality. It has been demonstrated that in all stages of CKD, there is a negative correlation between IS and PCS levels and the estimated glomerular filtration rate (eGFR) [3]. Similarly, TMAO has been associated with an increased risk of mortality and cardiovascular events [4].

As our understanding of the gut microbiome’s role in the progression of CKD improves, addressing dysbiosis becomes a primary therapeutic target. While dietary interventions remain an initial strategy for reducing uremic toxins, most patients struggle to adhere to the recommended diet in the long term [5]. As a result, there is growing interest in evaluating the efficacy of probiotics, prebiotics, and fecal microbial transplants (FMTs) as potential therapeutic strategies for CKD. This review article will provide an overview of the current literature on probiotics, prebiotics, synbiotics, and FMT and discuss their potential application for the management of CKD.

## 2. Probiotics

Probiotics are essential in promoting gut health by regulating the immune response, maintaining intestinal microbial balance, and creating an unfavorable environment for pathogenic bacteria to thrive. The precise mechanisms of action depend on the specific probiotic strain and may involve producing antibacterial compounds such as lactic acid, hydroperoxides, and bacteriocins blocking epithelial cell binding sites, upregulating tight junction molecules, degrading toxin receptors, modifying pH, and competing for nutrients [6,7]. Commonly used microorganisms in probiotic preparations include bacterial genera such as Lactobacillus, Bifidobacterium, Escherichia, Enterococcus, and Bacillus and the fungal genus Saccharomyces [7]. They can be administered in pockets to be dissolved in water or milk, as capsules, or added to yogurt. 

In CKD patients, the most commonly administered probiotics are *Bifidobacteria longum*, *B. bifidum*, *Lactobacillus acidophilus*, *L. casei*, *L. sakei*, *L. reuteri*, and *Streptococcus thermophilus.* Because Bifidobacteria and Lactobacillus are effective in reducing inflammation in autoimmune conditions, they have been heavily targeted to modulate the deleterious effects of CKD [8]. In particular, Bifidobacteria are capable of producing vital vitamins and stimulating the growth of another beneficial bacterium such as *Lactobacillus* [9]. Investigators have used cocktails with abundant Bifidobacteria and Lactobacilli, accounting for as much as 80% of the mix [2]. It is worth mentioning that certain strains such as *S. thermophilus* KB 19, *L. acidophilus* KB 27, and *B. longum* KB 31 did not exhibit any benefits in CKD patients [2].

The use of probiotics in the early stages of CKD, stages 1–2, is limited due to the lack of data characterizing the microbiota profile. The majority of studies have evaluated the use of probiotics in CKD stages 3–5 [2]. These investigations have demonstrated an overwhelming positive influence observed both in animals and humans [2,10,11,12]. A large cross-sectional study of 888 CKD patients reported that consumption of probiotic yogurt significantly reduced C-reactive protein levels [13]. Likewise, a meta-analysis of 13 trails that included 671 CKD patients reported a significant decrease in the total PCS level, inflammatory markers, blood urea nitrogen, creatinine, oxidative stress, and fecal pH with the use of probiotics [13]. Interestingly, a randomized control trial (RCT) has utilized a combination of probiotics and a low-protein diet in CKD stage 4 patients [14]. This study found that there is a reduction in PCS, IS, lipoprotein-associated phospholipase A2, and total cholesterol with the intervention group of a low-protein diet and probiotics. Furthermore, patients in the intervention group exhibited a reduction in the progression of ESRD and initiation of dialysis.

In hemodialysis (HD) patients, the use of probiotics has had mixed results due to variations in probiotic interventions and end-points. Individual studies have reported a decrease in PCS or IS, but not both [15,16]. However, a metanalysis showed an overall benefit of probiotics in reducing PCS, biomarkers of inflammation, and oxidative stress [17]. Additionally, the cardiovascular effects of probiotics on HD patients have yet to be established. A potential utility of probiotics for HD patients is its effectiveness in reducing gastrointestinal symptoms (diarrhea, abdominal pain, constipation). These symptoms are not uncommon in this population, occurring in 32–85% of HD patients [18]. Of interest, probiotic administration in patients on peritoneal dialysis (PD) showed a significant decrease in proinflammatory cytokines and endotoxins and an increase in anti-inflammatory cytokines, particularly IL-10 [15,16]. In one study, six months of probiotics supplementation was reported to preserve and maintain residual renal function, although the levels of urea, creatinine, and uric acid remained unchanged [19]. Whether PD patients benefit more than HD patients is unclear. Nevertheless, no adverse events were noted with the use of probiotics in HD or PD patients. 

### Takeaway

Probiotics, particularly Bifidobacteria and Lactobacillus strains, have shown promise in modulating the deleterious effects of chronic kidney disease (CKD) by reducing inflammation. They have been widely studied in CKD stages 3–5, with positive outcomes observed in both animal and human studies. Consumption of probiotic yogurt has been found to significantly reduce inflammatory markers in CKD patients, and a meta-analysis reported improvements in various parameters including PCS levels, oxidative stress, and fecal pH. However, the use of probiotics in HD patients has yielded mixed results, with variations in interventions and outcomes. Probiotics may be effective in reducing gastrointestinal symptoms in HD patients, but their cardiovascular effects and potential benefits in PD patients require further investigation. Nonetheless, no adverse events have been reported with probiotic use in HD or PD patients.

Overall, the preliminary data have shown a reduction in toxic and inflammatory components in CKD stage 3–5 patients supplemented with probiotics. Yet, it is unclear whether these changes can impact and halt the progression of CKD. To better understand the direct relationship between probiotics and CKD, kidney function needs to be measured. Additional studies with an appropriate sample size and follow-up are needed to conclusively establish the positive impact of probiotics on reducing CKD progression.

## 3. Prebiotics

Prebiotics are food ingredients that cannot be digested. They provide a beneficial effect on the host by encouraging the growth and activity of particular bacteria in the colon [8]. They are made up of polymer bonds, which makes them resistant to gastric breakdown. Prebiotic consumption has been linked to immune regulation, pathogen resistance, intestinal barrier function, increased mineral absorption, and reduced blood lipid levels [8]. One of their key functions is to promote bacteria that utilize short-chain fatty acids (SCFAs) to maintain intestinal and overall health. Prebiotics are found in more than 36,000 plant-based foods, such as artichokes, asparagus, chicory, garlic, onions, wheat, and bananas, and can also be added to food for health benefits [8]. The most common prebiotics include inulin, glucooligosaccharides, lactulose, oligofructose, galactose derivatives, and β-glucans [8].

Preliminary animal studies demonstrated a decrease in uremic toxins with prebiotic supplementation [20]. However, conflicting data have been reported regarding a reduction in inflammatory markers in human subjects with CKD stages 1–5 [20]. One study found no significant difference in IS and IAA concentrations in patients receiving prebiotics, whereas others reported varied effects of PCS and IS following prebiotic administration [15,21,22]. In stark contrast, an elegant meta-analysis conducted on 25 studies revealed that prebiotics were more effective than probiotics in reducing IS, malondialdehyde, Interleukin-6, and tumor necrosis factor-alpha in ESRD patients [23]. A possible explanation for this finding is that prebiotics increase SCFAs, which reduces reactive oxygen species, inflammation, and fibrosis in the kidney [23].

### Takeaway

While the data on the effects of prebiotics in CKD patients are still conflicting, preliminary animal studies suggest a decrease in uremic toxins with prebiotic supplementation. Some studies in human subjects have reported varying effects on inflammatory markers, but a meta-analysis found that prebiotics were more effective than probiotics in reducing inflammation in ESRD patients. This may be attributed to the ability of prebiotics to increase short-chain fatty acids, which can reduce oxidative stress, inflammation, and kidney fibrosis. At present, the role of prebiotics in CKD stages 1–5 remains uncertain. However, there is greater clarity for patients undergoing hemodialysis, where prebiotics are beneficial. 

## 4. Synbiotics

Synbiotics are a blend of prebiotics and probiotics that have a synergistic effect. Prebiotics can stimulate probiotics to regulate intestinal metabolism, maintain biostructure, and develop beneficial microbiota while inhibiting pathogens [24]. Additionally, synbiotics decrease unwanted metabolite concentrations, inactivate cancerogenic substances, and increase the levels of health-promoting compounds such as short-chain fatty acids, ketones, carbon disulfides, and methyl acetates [24].

Multiple studies have documented a positive impact of synbiotics on reducing CK progression. The SYNERGY II trial was conducted to determine the long-term effects of symbiotic supplementation in patients with moderate-to-severe CKD [25]. The results revealed that the symbiotic group showed a considerable decline in eGFR and a rise in serum creatinine compared with the placebo group. Inadequate statistical power and sample size were some of the limitations of this study. This finding contradicted previous short-term studies, which showed no effect of symbiotic supplementation on eGFR or serum creatinine [25,26]. Moreover, SYNERGY II showed no effect on uremic toxins, IS, and PCS. These findings are consistent with a meta-analysis of five studies of symbiotic supplementation [27]. Three of these studies were conducted in CKD stage 3 and 4 patients [25,26,28], while the other two were conducted in hemodialysis patients [29,30]. Yet another meta-analysis of synbiotics in hemodialysis patients showed that they are superior to prebiotics and probiotics in reducing CRP levels and endotoxins [23]. 

### Takeaway

While the long-term effects of synbiotics in CKD are still under investigation, studies have demonstrated mixed results. The SYNERGY II trial reported a decline in eGFR and an increase in serum creatinine in the symbiotic group compared with the placebo group, contrary to previous short-term studies. However, there was no significant impact on uremic toxins. Meta-analyses have suggested that synbiotics may be more effective than prebiotics and probiotics in reducing CRP levels and endotoxins in hemodialysis patients. Further research is needed to determine the optimal formulations, dosages, and duration of synbiotic interventions in CKD and to address the inconsistencies in the existing findings.

The SYNERGY II trial [24] has raised serious concerns about the long-term use of synbiotics, as it is the first study to show a potential reduction in kidney function. However, the study was underpowered. Based upon the current literature, symbiotic use cannot be endorsed to halt CKD. 

## 5. Fecal Microbiota Transplant 

FMT, or fecal microbiota transplant, is a medical procedure that involves transferring fecal matter from a healthy donor into the digestive tract of a patient with a diseased gut to restore the balance of the gut microbiota. It gained popularity after the United States Food and Drug Administration (FDA) approved FMT for refractory Clostridium difficile infection in 2013 [31]. FMT is now being investigated and used for a wider range of additional indications including inflammatory bowel disease, metabolic syndrome, autoimmune disorders, idiopathic thrombocytopenic purpura, and numerous other conditions [31]. The fecal material can be administered through various methods such as the upper gastrointestinal (GI) route (using esophagogastroduodenoscopy (EGD) or a nasogastric, nasojujunal, or nasoduodenal tube), lower GI route (colonoscopy or retention enema), or via oral capsules [31]. FMT modulates the immune system to reduce inflammation and restore gut homeostasis by activating immunoregulatory signaling pathways [32]. This increases IL-10 secretion by innate and adaptive immune cells while inhibiting antigen processing and presentation by monocytes, dendritic cells, and macrophages in the intestine [32].

The majority of FMT studies in CKD are conducted in animal models. When FMT was conducted from rodents with CKD to healthy rodents, there was an increase in uremic toxins, inflammation, oxidative stress, and interstitial fibrosis in the healthy rodents [33]. FMT experiments in mice with diabetic kidney disease yielded similar results, suggesting that the variations in the gut microbiome could be a contributing factor to the severity of kidney disease [32]. Similarly, there is overwhelming evidence of a significant reduction in uremic toxins and inflammation and an overall improvement in kidney function in CKD rodents with FMT from healthy donors [32]. One study distinguishes itself in creating a selective cocktail of microbials (*Escherichia coli*, *Bacillus subtilis*, and *Lactobacillus acidophilus*) to transfer, instead of the whole fecal matter [10]. Of interest, this study also demonstrated a significant reduction in serum urea and creatinine levels in both acute kidney injury (AKI) and CKD rodents [10]. 

**Figure 1:**
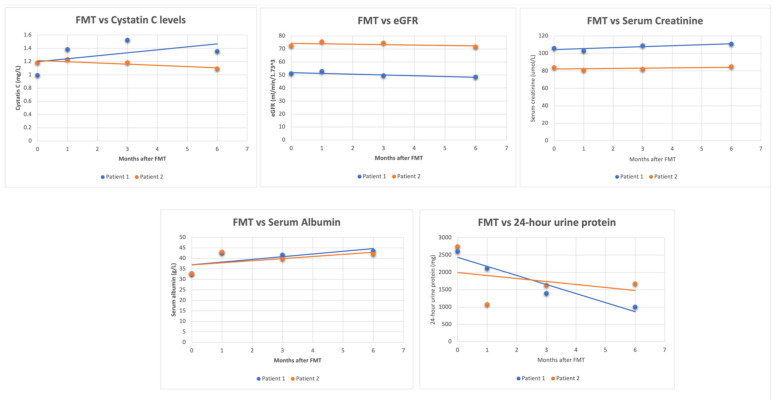
Graphs collectively showing the changes in these parameters after undergoing FMT for IgAN.

Three cases involving the use of FMT in patients with kidney disease have been reported [34,35]. In two cases of IgA nephropathy, changes in laboratory values were observed up to six months after FMT [35], whereas one case of MN reported immediate changes in laboratory values following FMT [34]. Figure 1 illustrates the lab trends of the two IgAN cases. At 6 months post-FMT, there was an average decrease of 50.3% in 24 h urine protein, a 3.2% decrease in eGFR, a 2.9% increase in creatinine, and a 30.88% increase in serum albumin. The significant decline in 24 h urine protein is noteworthy because patients with sustained proteinuria of >1 g/day are likely to undergo a faster decline in renal function and develop ESRD [36]. In the case of MN, the immediate short-term results were similar, except for a decrease in creatinine. Due to differences in the FMT method and follow-up period, comparing the three cases side by side is challenging. Nonetheless, both cases highlight the potential short-term and long-term benefits of FMT.

### Takeaway

Animal studies have demonstrated the potential of FMT in improving kidney function and reducing uremic toxins, inflammation, and oxidative stress. Limited clinical reports in patients with kidney disease have shown promising results with FMT, including reductions in proteinuria and improvements in laboratory values. Although the three cases demonstrating the benefits of FMT in CKD are overwhelmingly positive, the widespread use of FMT for CKD patients needs further exploration in human studies. FMT benefits set aside, the safety-profile needs to be thoroughly investigated. It is also important to note that a reduction in the toxic and inflammatory components does not necessarily mean that there is a reduction in CKD progression. Therefore, studies should focus on measuring kidney function. Furthermore, the statement, “one stool does not fit all” [37] emphasizes the need to individualize the microbial profile in fecal transplant in consideration of the patient’s current gut biome. The promising results obtained by the three human cases thus far underscore the urgency to evaluate the role of FMT in CKD. 

## 6. Conclusions

In conclusion, the gut microbiome has a crucial role in regulating various physiological functions, including the immune system, fat metabolism, and the synthesis of essential vitamins and amino acids. Dysbiosis has been linked to numerous immune-mediated conditions, including CKD, which is exacerbated by the retention of metabolic waste products and uremic toxins derived from gut bacteria. Probiotics, prebiotics, synbiotics, and FMT are emerging therapies that show promise in restoring gut homeostasis in CKD patients. Probiotics, in particular, have shown positive outcomes in reducing inflammation, oxidative stress, and uremic toxin levels in CKD patients. FMT, though still in its early stages of investigation, is a promising therapeutic option for CKD patients with severe dysbiosis. Further studies are urgently needed to optimize the composition of probiotics, prebiotics, and synbiotics, as well as the FMT protocol, to maximize their efficacy and safety in CKD patients. Overall, these therapies can potentially be used in synergy with conventional therapies and may hold the key to managing CKD and improving patient outcomes.

## Figures and Tables

**Table 1:** Summarization of the mechanisms of how dysbiosis can promote CKD [2].

Mechanism	Elaboration of How These Mechanisms Can Promote CKD
Inappropriate activation of the immune system	Treg/Th17 imbalance: Increase in Th17 cells and decrease in Tregs, leading to increased secretions of IL-17 and other proinflammatory cytokines. These Th17 cells also accumulate in the kidneys.
Gut-microbiota-derived metabolites	1. Increased uremic toxins (TMAO, IS, PCS): induce inflammation and promote tubulointerstitial fibrosis and glomerulosclerosis. 2. Reduction in SCFAs (acetate, propionate, butyrate): activation of G-protein-coupled receptors to promote reactive oxygen species triggers hypertension through interaction with renal afferent arterioles and peripheral blood vessels. 3. Increased endotoxins: promotes systemic inflammation, cardiac injury, and atherosclerosis.
Activation of Renin–Angiotensin System (RAS)	Activation of RAS by uremic toxins through proinflammatory transforming growth factor-Beta 1 (TGF-B1).
Disruption of the intestinal barrier	Degradation of colonic tight junctions through a reduction in SCFAs and increase in uremic toxins.

## Data Availability

Not Applicable.

## References

[1] Liu Y., Alookaran J., Rhoads J. (2018). Probiotics in Autoimmune and Inflammatory Disorders. Nutrients.

[2] Tian N., Li L., Ng J.K.C., Li P.K.T. (2022). The Potential Benefits and Controversies of Probiotics Use in Patients at Different Stages of Chronic Kidney Disease. Nutrients.

[3] Holle J., Querfeld U., Kirchner M., Anninos A., Okun J., Thurn-Valsassina D., Bayazit A., Niemirska A., Canpolat N., Bulut I.K. (2019). Indoxyl sulfate associates with cardiovascular phenotype in children with chronic kidney disease. Pediatr. Nephrol..

[4] Jiang S., Xie S., Lv D., Zhang Y., Deng J., Zeng L., Chen Y. (2016). A reduction in the butyrate producing species Roseburia spp. and *Faecalibacterium prausnitzii* is associated with chronic kidney disease progression. Antonie Van Leeuwenhoek.

[5] MacLaughlin H.L., Sarafidis P.A., Greenwood S.A., Campbell K.L., Hall W.L., Macdougall I.C. (2012). Compliance With a Structured Weight Loss Program Is Associated With Reduced Systolic Blood Pressure in Obese Patients With Chronic Kidney Disease. Am. J. Hypertens..

[6] Veerappan G.R., Betteridge J., Young P.E. (2012). Probiotics for the Treatment of Inflammatory Bowel Disease. Curr. Gastroenterol. Rep..

[7] Fijan S. (2014). Microorganisms with Claimed Probiotic Properties: An Overview of Recent Literature. Int. J. Environ. Res. Public Health.

[8] Slavin J. (2013). Fiber and prebiotics: Mechanisms and health benefits. Nutrients.

[9] Fagundes R.A.B., Soder T.F., Grokoski K.C., Benetti F., Mendes R.H. (2018). Probiotics in the treatment of chronic kidney disease: A systematic review. J. Bras. Nefrol..

[10] Zheng D.W., Pan P., Chen K.W., Fan J.X., Li C.X., Cheng H., Zhang X.Z. (2020). An orally delivered microbial cocktail for the removal of nitrogenous metabolic waste in animal models of kidney failure. Nat. Biomed. Eng..

[11] Chen L., Zhang S., Wu S., Ren Z., Liu G., Wu J. (2021). Synergistic Protective Effect of Konjac Mannan Oligosaccharides and *Bacillus subtilis* on Intestinal Epithelial Barrier Dysfunction in Caco-2 Cell Model and Mice Model of Lipopolysaccharide Stimulation. Front. Immunol..

[12] Hernández-Chirlaque C., Aranda C.J., Ocón B., Capitán-Cañadas F., Ortega-González M., Carrero J.J., Suárez M.D., Zarzuelo A., Sanchez de Medina F., Martínez-Augustin O. (2016). Germ-free and Antibiotic-treated Mice are Highly Susceptible to Epithelial Injury in DSS Colitis. J. Crohns Colitis.

[13] Zheng H.J., Guo J., Wang Q., Wang L., Wang Y., Zhang F., Huang W.J., Zhang W., Liu W.J., Wang Y. (2021). Probiotics, prebiotics, and synbiotics for the improvement of metabolic profiles in patients with chronic kidney disease: A systematic review and meta-analysis of randomized controlled trials. Crit. Rev. Food Sci. Nutr..

[14] De Mauri A., Carrera D., Bagnati M., Rolla R., Vidali M., Chiarinotti D., Pane M., Amoruso A., Del Piano M. (2022). Probiotics-Supplemented Low-Protein Diet for Microbiota Modulation in Patients with Advanced Chronic Kidney Disease (ProLowCKD): Results from a Placebo-Controlled Randomized Trial. Nutrients.

[15] Meijers B.K.I., De Preter V., Verbeke K., Vanrenterghem Y., Evenepoel P. (2010). p-Cresyl sulfate serum concentrations in haemodialysis patients are reduced by the prebiotic oligofructose-enriched inulin. Nephrol. Dial. Transplant..

[16] Pan Y., Yang L., Dai B., Lin B., Lin S., Lin E. (2021). Effects of Probiotics on Malnutrition and Health-Related Quality of Life in Patients Undergoing Peritoneal Dialysis: A Randomized Controlled Trial. J. Ren. Nutr..

[17] Nguyen T.T.U., Kim H.W., Kim W. (2021). Effects of Probiotics, Prebiotics, and Synbiotics on Uremic Toxins, Inflammation, and Oxidative Stress in Hemodialysis Patients: A Systematic Review and Meta-Analysis of Randomized Controlled Trials. J. Clin. Med..

[18] Tang W.W., Wang Z., Kennedy D.J., Wu Y., Buffa J.A., Agatisa-Boyle B., Li X.S., Levison B.S., Hazen S.L. (2015). Gut Microbiota-Dependent Trimethylamine N -Oxide (TMAO) Pathway Contributes to Both Development of Renal Insufficiency and Mortality Risk in Chronic Kidney Disease. Circ. Res..

[19] Wang I.K., Wu Y.Y., Yang Y.F., Ting I.W., Lin C.C., Yen T.H., Chen J.H., Wang C.H., Huang C.C., Lin H.C. (2015). The effect of probiotics on serum levels of cytokine and endotoxin in peritoneal dialysis patients: A randomised, double-blind, placebo-controlled trial. Benef. Microbes.

[20] Salmean Y.A., Segal M.S., Palii S.P., Dahl W.J. (2015). Fiber supplementation lowers plasma p-cresol in chronic kidney disease patients. J. Ren. Nutr..

[21] Sirich T.L., Plummer N.S., Gardner C.D., Hostetter T.H., Meyer T.W. (2014). Effect of Increasing Dietary Fiber on Plasma Levels of Colon-Derived Solutes in Hemodialysis Patients. Clin. J. Am. Soc. Nephrol..

[22] Manigandan T., Mangaiyarkarasi S.P., Hemalatha R., Hemalatha V.T., Murali N.P. (2012). Probiotics, Prebiotics and Synbiotics—A Review. Biomed. Pharmacol. J..

[23] Bres E., Koppe L. (2019). Is there still a place for prebiotics in chronic kidney disease?. Nephrol. Dial. Transplant..

[24] McFarlane C., Krishnasamy R., Stanton T., Savill E., Snelson M., Mihala G., Kelly J.T., Morrison M., Johnson D.W., Campbell K.L. (2021). Synbiotics Easing Renal Failure by Improving Gut Microbiology II (SYNERGY II): A Feasibility Randomized Controlled Trial. Nutrients.

[25] Rossi M., Johnson D.W., Morrison M., Pascoe E.M., Coombes J.S., Forbes J.M., Szeto C.C., McWhinney B.C., Ungerer J.P., Campbell K.L. (2016). Synbiotics Easing Renal Failure by Improving Gut Microbiology (SYNERGY). Clin. J. Am. Soc. Nephrol..

[26] Dehghani H., Heidari F., Mozaffari-Khosravi H., Nouri-Majelan N., Dehghani A. (2016). Synbiotic Supplementations for Azotemia in Patients With Chronic Kidney Disease: A Randomized Controlled Trial. Iran. J. Kidney Dis..

[27] McFarlane C., Ramos C.I., Johnson D.W., Campbell K.L. (2019). Prebiotic, Probiotic, and Synbiotic Supplementation in Chronic Kidney Disease: A Systematic Review and Meta-analysis. J. Ren. Nutr..

[28] Guida B., Germanò R., Trio R., Russo D., Memoli B., Grumetto L., Barbato F., Cataldi M. (2014). Effect of short-term synbiotic treatment on plasma p-cresol levels in patients with chronic renal failure: A randomized clinical trial. Nutr. Metab. Cardiovasc. Dis..

[29] Cruz-Mora J., Martínez-Hernández N.E., del Campo-López F.M., Viramontes-Hörner D., Vizmanos-Lamotte B., Muñoz-Valle J.F., García-García G., Parra-Rojas I., Castro-Alarcón N. (2014). Effects of a Symbiotic on Gut Microbiota in Mexican Patients With End-Stage Renal Disease. J. Ren. Nutr..

[30] Viramontes-Hörner D., Márquez-Sandoval F., Martín-del-Campo F., Vizmanos-Lamotte B., Sandoval-Rodríguez A., Armendáriz-Borunda J., García-Bejarano H., Renoirte-López K., García-García G. (2015). Effect of a Symbiotic Gel (Lactobacillus acidophilus + Bifidobacterium lactis + Inulin) on Presence and Severity of Gastrointestinal Symptoms in Hemodialysis Patients. J. Ren. Nutr..

[31] Vindigni S.M., Surawicz C.M. (2017). Fecal Microbiota Transplantation. Gastroenterol. Clin. N. Am..

[32] Bian J., Liebert A., Bicknell B., Chen X.M., Huang C., Pollock C.A. (2022). Faecal Microbiota Transplantation and Chronic Kidney Disease. Nutrients.

[33] Wang X., Yang S., Li S., Zhao L., Hao Y., Qin J., Zhang L., Zhang C., Bian W., Zuo L.I. (2020). Aberrant gut microbiota alters host metabolome and impacts renal failure in humans and rodents. Gut.

[34] Zhou G., Zeng J., Peng L., Wang L., Zheng W., Yang Y. (2021). Fecal microbiota transplantation for membranous nephropathy. CEN Case Rep..

[35] Zhao J., Bai M., Yang X., Wang Y., Li R., Sun S. (2021). Alleviation of refractory IgA nephropathy by intensive fecal microbiota transplantation: The first case reports. Ren. Fail..

[36] Le W., Liang S., Hu Y., Deng K., Bao H., Zeng C., Liu Z. (2012). Long-term renal survival and related risk factors in patients with IgA nephropathy: Results from a cohort of 1155 cases in a Chinese adult population. Nephrol. Dial. Transplant..

[37] Danne C., Rolhion N., Sokol H. (2021). Recipient factors in faecal microbiota transplantation: One stool does not fit all. Nat. Rev. Gastroenterol. Hepatol..

